# Ferulic Acid Ameliorates Hepatic Inflammation and Fibrotic Liver Injury by Inhibiting PTP1B Activity and Subsequent Promoting AMPK Phosphorylation

**DOI:** 10.3389/fphar.2021.754976

**Published:** 2021-09-08

**Authors:** Jianzhi Wu, Xiaoyong Xue, Guifang Fan, Yiqing Gu, Fei Zhou, Qi Zheng, Runping Liu, Yajing Li, Boning Ma, Shuo Li, Guangrui Huang, Lin Ma, Xiaojiaoyang Li

**Affiliations:** ^1^ School of Life Sciences, Beijing University of Chinese Medicine, Beijing, China; ^2^ School of Chinese Materia Medica, Beijing University of Chinese Medicine, Beijing, China

**Keywords:** ferulic acid, AMPK, PTP1B, oxidative stress, inflammation, liver fibrosis

## Abstract

Chronic inflammation in response to persistent exogenous stimuli or damage results in liver fibrosis, which subsequently progresses into malignant liver diseases with high morbidity and mortality. Ferulic acid (FA) is a phenolic acid widely isolated from abundant plants and exhibits multiple biological activities including anti-oxidant, anti-inflammation and enhancement of immune responses. Adenosine monophosphate-activated protein kinase (AMPK) functions as a critical energy sensor and is regulated through the phosphorylation of liver kinases like LKB1 or dephosphorylation by protein tyrosine phosphatases (PTPs). However, the role of FA in carbon tetrachloride (CCl_4_)-induced chronic inflammation and liver fibrosis and AMPK activation has not been elucidated. Here we reported that FA ameliorated CCl_4_-induced inflammation and fibrotic liver damage in mice as indicated by reduced levels of serum liver function enzyme activities and decreased expression of genes and proteins associated with fibrogenesis. Additionally, FA inhibited hepatic oxidative stress, macrophage activation and HSC activation via AMPK phosphorylation in different liver cells. Mechanically, without the participation of LKB1, FA-induced anti-inflammatory and anti-fibrotic effects were abrogated by a specific AMPK inhibitor, compound C. Combining with the results of molecular docking, surface plasmon resonance and co-immunoprecipitation assays, we further demonstrated that FA directly bound to and inhibited PTP1B, an enzyme responsible for dephosphorylating key protein kinases, and eventually leading to the phosphorylation of AMPK. In summary, our results indicated that FA alleviated oxidative stress, hepatic inflammation and fibrotic response in livers through PTP1B-AMPK signaling pathways. Taken together, we provide novel insights into the potential of FA as a natural product-derived therapeutic agent for the treatment of fibrotic liver injury.

## Introduction

Liver fibrosis is a dynamic process leading to inflammatory cascades, excessive deposition of extracellular matrix (ECM) and the formation of fibrous scars. With persistent inflammatory stimuli or exogenous damage, liver fibrosis is becoming a leading cause of cirrhosis and hepatocellular carcinoma with high morbidity and mortality (Kisseleva, 2017). Underlying etiologies in fibrotic liver injury comprise but not limited to viral infection, alcoholic or nonalcoholic steatohepatitis, oxidative stress and exogenous noxious stimuli (Parola and Pinzani, 2019). Meanwhile, liver fibrosis is not a single-target disease and its progression is attributed to the highly active crosstalk between several liver cell types, including hepatocytes, macrophages and hepatic stellate cells (HSCs). Although liver fibrosis is theoretically reversible, numerous attempts have failed due to a narrow therapeutic window, limited therapeutic efficacy or undesired severe adverse effects.

Adenosine monophosphate-activated protein kinase (AMPK), an obligate heterotrimer composed of three subunits (α, β, and γ), is regarded as a critical cellular energy sensor that controls energy expenditure and storage. Besides the reduced adenosine triphosphate (ATP)/adenosine diphosphate (ADP) ratio caused by any cellular stress (Li et al., 2015), the phosphorylation of extracellular-signal-regulated protein kinase 1/2 (ERK1/2) and liver kinase B1 (LKB1) are the major upstream signal cascades of AMPK activation (Li et al., 2017). It has long been conceived that AMPK is critical for maintaining normal hepatic physiological functions and its activity is decreased during several liver diseases. The activation of AMPKα1 upregulated cyclin A2 transcription, promoted hepatocyte proliferation and eventually restored the liver mass after partial hepatectomy (Merlen et al., 2014). On the other side, once the activation of AMPK was decreased, pro-apoptotic caspase-6 cleaved Bid to induce cytochrome c release that led to hepatocyte damage and fibrotic liver injury (Zhao et al., 2020). Most recently, Qian Lin et al. constructed liver-specific AMPK knockout mice and investigated the specific role of phosphorylated AMPK in fibrotic steatohepatitis. The hepatic depletion of AMPK completely disrupted hepatic lipid metabolism, triggered inflammatory response and aggregated liver fibrosis in a western diet-induced liver steatotic mouse model (Lin et al., 2020). Collectively, considering the vital role that AMPK plays in the progression of fibrotic liver injury, the identification of novel drugs targeting AMPK is urgently needed.

Mechanisms involved in AMPK activation include activating upstream kinases of AMPK or inactivating dephosphorylated kinases that control the activation of AMPK. The aberrant interplay between protein-tyrosine phosphatases (PTPs), responsible for protein dephosphorylation, and tyrosine kinases affects the function of multiple proteins, disrupts the normal liver function and impacts the progression of chronic liver diseases (Rubio et al., 2020). PTP1B is one of the most critical members of PTPs family and expresses in multiple hepatic cells. Studies revealed that PTP1B has become an effective target for the therapeutic intervention of hepatic injury and lipid metabolism disorders (Mobasher et al., 2013; Hu et al., 2021). Recently, researches concerning the role of PTP1B in fibrotic liver injury have stepped into a new stage. Specific liver-knockout of PTP1B protected mice from chronic alcohol plus binge-induced oxidative stress, liver fibrosis and inflammation *via* the inhibition of nuclear factor kappa B (NF-κB) and reduction of nicotinamide adenine dinucleotide phosphate oxidase 2 (NOX2) and NOX4 expression (Hsu et al., 2020). Consistent with prior findings, PTP1B overexpression notably hindered the inactivation of HSC-T6 cells, as manifested by restoring the levels of collagen 1 (COL1) and alpha-smooth muscle actin (α-SMA) (Chen et al., 2016). However, whether hepatic PTP1B influences hepatic inflammation and liver fibrosis through the phosphorylation of AMPK and potential PTP1B inhibitors are suitable for treating fibrotic liver injury remain to be identified.

Ferulic acid (3-methoxy-4-hydroxycinnamic acid, FA) is a phenolic acid widely distributed in abundant grains, vegetables and plants such as *Angelicae Sinensis Radix*, *Ligusticum Chuanxiong Rhizoma* and *Cimicifuga racemose* (Oliveira et al., 2019). It also exhibits a broad spectrum of biological activities, including repairing mitochondrial dysfunction (Luo et al., 2020), anti-oxidant (Xie et al., 2020), enhancement of immune function (Kohno et al., 2020) and preventing epithelial-mesenchymal transition (Ali et al., 2021). Several recent studies further provided evidence for the hepatoprotective effects of FA. Administration of FA improved lipid metabolism and hepatic inflammation in apolipoprotein E-deficient mice fed with high fat diet (HFD) by upregulating AMPKα and downregulating lipogenic genes (Gu et al., 2021). FA also offered significant therapeutic benefits for oxidative stress and liver injury depending on its ability to activate Nrf2/HO-1 and PPARγ pathways (Mahmoud et al., 2020). In addition, FA significantly alleviated septic liver injury through the GSK-3β/NF-κB/CREB pathway both *in vivo* and *in vivo* (Cao et al., 2021). Moreover, it could prevent acetaminophen-induced inflammatory response in livers through the inhibition of TLR4/NF-κB signalings (Yuan et al., 2016). Up to now, relevant studies only preliminarily verified the protective effects of FA using western diet- or toxins-induced mouse models but was still lack of in-depth investigation on mechanism (Mu et al., 2018; Gu et al., 2021). Therefore, the protective effects of FA against fibrotic liver injury and the potential mechanisms involved need to be clarified and is the purpose of this study.

In the current study, we inspected the effects of FA on carbon tetrachloride (CCl_4_)-induced fibrotic mouse model and in different drugs-stimulated liver cells. Our findings indicated that FA prevented all histological alterations, suppressed hepatic oxidative stress, inflammatory response, macrophage activation and HSC activation by phosphorylating AMPK. We also demonstrated that FA directly bound to PTP1B, suppressed its enzyme activity rather than its expression and subsequently contributed to AMPK activation.

## Materials and Methods

### Materials

FA and methyl ferulate (MF) were purchased from Innochem Technology Co., Ltd. (Beijing, China). Compound C (CC) was purchased from Selleck Chemicals (Shanghai, China). CCl_4_ and all cell culture supplemental components were purchased from Sigma-Aldrich (St. Louis, United States). HiScript III RT SuperMix cDNA reverse transcription Kits (R323-01) and AceQ^TM^ Universal SYBR qPCR Master Mix (Q511-02) were obtained from Vazyme Biotech (Nanjing, China). Antibodies against p-ERK1/2 (sc-7383), ERK1 (sc-271269), ERK2 (sc-81457), LKB1 (sc-32245) were purchased from Santa Cruz Biotechnology (Santa Cruz, United States). Antibodies against p-AMPK (ab23875), AMPK (ab131512) and p-LKB1 (ab63473) were from Abcam (Cambridge, United States). Antibodies against ALB (4929S), β-actin (4970S) and normal rabbit IgG (2729S) were obtained from Cell Signaling Technology (Danvers, United States). Antibodies against PTP1B (11334-1-AP) and FIBRONENCTIN (FN) (15613-1-AP) were purchased from Proteintech Group, Inc (Rosemont, United States). Goat anti-rabbit IgG-HRP (abs20040) and goat anti-mouse IgG-HRP (abs20039) were obtained from Absin Bioscience (Shanghai, China). Protein A/G-PLUS agarose beads (sc-2003) was purchased from Santa Cruz Biotechnology (Santa Cruz, United States).

### Isolation and Culture of Mouse Primary Hepatocytes

Mouse primary hepatocytes (MPHs) were isolated according to a method of two-step collagenase digestion and seeded into collagen pre-coated cell dishes as previously described (Li et al., 2018). After 4 h attachment, MPHs were cultured with William’s E medium supplemented with penicillin G (100 U/ml), streptomycin (100 μg/ml), dexamethasone (0.1 μM) and thyroxine (0.1 μM) for further experiments.

### Cell Culture and Treatment

RAW 264.7 cells and LX-2 cells were obtained from ATCC and cultured with Dulbecco’s modified Eagle medium (DMEM) supplemented with penicillin G (100 U/ml), streptomycin (100 μg/ml) and 10% fetal bovine serum (FBS) in the atmosphere of 5% CO_2_ at 37°C. After seeded, RAW 264.7 cells were treated with FA or MF (both 100 μM) for 0.5, 1, 2, 4 and 6 h for the time course experiment or treated with FA or MF (both 50, 100, and 200 μM) for 24 h. LX-2 cells and MPHs were either treated with FA or MF (25 μM) at different time points or treated with FA or MF (both 12.5, 25 and 50 μM) for 24 h. To explore the anti-fibrotic effects of FA, LX-2 cells were pretreated with FA (25 μM) for 1 h and then treated with transforming growth factor-beta (TGF-β) (5 ng/ml) for another 2 h or 24 h. To examine the anti-inflammatory activity of FA, RAW 264.7 cells were administrated with 100 μM FA for 1 h, followed by 100 ng/ml lipopolysaccharide (LPS) treatment for another 4 h. For the AMPK inhibition study, after 10 μM CC-pretreated, MPHs, RAW 264.7 cells and LX-2 cells were treated with different dosages of FA. To investigate the hepatoprotective effects of FA, MPHs were pretreated with 25 μM FA for 1 h and then treated with 10 mM CCl_4_ for another 24 h.

### Animal Studies

C57BL/6J mice (22–24 g, male and female, SPF grade) were purchased from SIBEIFU Biotechnology Co, Ltd (Beijing, China). Mice were housed in a homoiothermic and sterile condition of 12-h light/dark cycles and fed standard chow and tap water at libitum. After 1 week acclimatization, mice were divided into five experimental groups (*n* = 6): 1) control group; 2) CCl_4_ group; 3) CCl_4_ + FA (low dose) group; 4) CCl_4_+ FA (medium dose) group; 5) CCl_4_+ FA (high dose) group. To chronically induce liver fibrosis, mice were received 1 ml/kg CCl_4_ by gavage twice a week for 8 weeks in groups (2–5) or were orally treated with the same volume of vehicle solution (olive oil) in group (1). To investigate the anti-fibrotic effects of FA, groups (3–5) were orally administrated with FA at different dosages (25, 50 and 100 mg/kg) for 4 weeks after 4 weeks of CCl_4_ administration. After treatment, mice were weighed, anesthetized with isoflurane and sacrificed to collect blood and livers. All animal studies and procedures were approved by the Institutional Animal Care and Use Committee of Beijing University of Chinese Medicine and were carried out in accordance with all guidelines and regulations.

### PTP1B Activity Assay

The activity of PTP1B was determined by measuring the free phosphate released from PTP1B substrate using the commercially available PTP1B Assay kit 539736) from Merck Millipore (Darmstadt, Germany). Different concentrations of FA and MF or the equal volume of assay buffer solution (as a negative control) were prepared and added in a 96-well plate, followed by incubation with PTP1B enzyme dilution and warmed substrate for 40 min at 30°C. After incubating wells for the desired duration, reactions were terminated with Red Reagent. The absorbance was measured at 620 nm wavelength by xMark™ Microplate Absorbance Spectrophotometer. The activity of PTP1B enzyme in different wells was calculated by the conversion of absorbance to phosphate concentration with the phosphate standard curve as follows.

$$\%\;Activity=\frac{\left[\text{FA or MF sample }\left(\text{nmol }po_{4}^{2-}\right)-“time\;0”\left(\text{nmol }po_{4}^{2-}\right)\right]}{\left[\text{Assay buffer }\left(\text{nmol }po_{4}^{2-}\right)-“time\;0”\left(\text{nmol }po_{4}^{2-}\right)\right]}\;\times \;100\text{\%}$$ (1)

### Histopathology, Masson’s Trichrome Staining and Immunohistochemistry

After immobilized with 4% formaldehyde, 4.5-μm paraffin sections were prepared and further stained with hematoxylin and eosin (H&E) and Masson’s Trichrome as previously described (Li et al., 2017). Cell damage and inflammatory infiltration in histological changes were determined by evaluating the degree of hepatic injury. H&E scoring was performed as previously described (Wu et al., 2021). Quantification of collagen deposition (the percentage of blue collagen area relative to the total staining area) in Masson’s Trichrome staining was conducted using ImageJ software. For IHC staining, paraffin sections were rehydrated, antigen retrieval by EDTA and blocked endogenous peroxidases with 0.3% H_2_O_2_. After blocked with BSA reagent supplemented with 10% goat serum, slides were incubated with primary antibody against FN (dilution, 1:100) at 4 °C overnight, washed and incubated with goat anti-mouse/rabbit IgG HRP polymer secondary antibody (ZSGB-BIO, Beijing, China). The images of staining sections were obtained by Aperio Versa (Leica, Wetzlar, Germany).

### Immunofluorescence Staining

After treatment, cells were rinsed with phosphate-buffered saline (PBS) buffer, fixed in 3.7% formaldehyde, blocked and permeabilized with 1% PBS-BSA with 0.1% Triton-X-100, followed by incubation with primary antibody against ALB (dilution, 1:500) in a wet chamber at 4 °C overnight. Later, cells were incubated with Alexa Fluor® 594 Goat anti-rat secondary antibody (dilution, 1:1,000) and then counterstained with DAPI. Immunofluorescence images were captured by Olympus FV3000 confocal laser scanning microscopy (Tokyo, Japan).

### Measurement of Intracellular ROS

A ROS assay kit (S0033S, Jiancheng Bioengineering Institute, Nanjing, China) with DCFH-DA as a fluorescent probe was used to determine the relative ROS levels in MPHs. After treatment, MPHs were washed with PBS and incubated with the 10 μmol/L of DCFH-DA probe in dark at 37°C for 30 min. After discarding the unreacted probes and washing twice with PBS, the fluorescent intensity of oxidized product was determined at 525 nm. Immunofluorescence images were captured by Olympus FV3000 confocal laser scanning microscopy (Tokyo, Japan).

### Molecular Docking Study

In silicon docking studies was performed using SYBYL-X 2.0 software (Tripos Inc, St. Louis, MO). The 3D structures of the compound FA and MF were downloaded from the ZINC database. The crystal structures of PTP1B (PDB ID:4I8N) in complex with an inhibitor of compound A [(4-{(2s)-2-(1,3-benzoxazol-2-yl)-2-[(4-fluorophenyl)sulfamoyl]ethyl}phenyl)amino](oxo)acetic acid and AMPK (PDB ID:6BX6) in complex with an inhibitor of compound B, SBI-0206965 were downloaded from RCSB protein data bank. The PTP1B and AMPK were optimized for molecular docking as follows. After removing the inhibitors and all of the water molecules and adding H atoms, side-chain amides were fixed and then prepared with the protein preparation module using default parameters. Furthermore, compound A, FA and MF were docked with the prepared PTP1B crystal structure, and compound B and FA were docked with AMPK by undertaking the Surflex-Dock (SFXC) docking mode. Meanwhile, the original inhibitors of compound A and compound B were respectively used to compare the interaction between residues and dispositions when PTP1B and AMPK were docked. Docking results were analyzed and visualized using SYBYL-X 2.0 software and Molecular Operating Environment software (Chemical Computing Group ULC, Canada).

### Co-immunoprecipitation Study

Protein A/G-PLUS agarose beads were incubated with PTP1B antibody or IgG in IP lysis buffer at 4 °C overnight. The next day, agarose beads were centrifuged and resuspended three times with IP buffer. After washing with ice-PBS, the cell pellet was gently lysed and centrifuged to collect the lysate for the following experiment. After pre-cleared by incubation with protein A/G-PLUS agarose beads for 1 h, cell lysate was incubated with PTP1B antibody- or lgG-coated beads on a 4 °C rotator overnight. After washed four times with IP buffer, the immunoprecipitated beads were denatured with 4x laemmli sample buffer (1610747, Bio-Rad) at 100°C, followed by western blot analysis to determine the binding capacity of PTP1B and p-AMPK or t-AMPK.

### Surface Plasmon Resonance Assay

The surface plasmon resonance (SPR) assay was conducted at 25°C using Biacore T200 SPR sensor (Biacore, GE Healthcare) with control software version 3.0. Briefly, a CM5 chip (carboxymethylated dextran surface) in Biacore PBS-EP running buffer was first activated following a standard EDC/NHS method. PTP1B protein was prepared in NaAc buffer (pH 4.5) to a final concentration of 20 μg/ml and was then injected into the channel of analysis instrument for 420 s followed by a 7 min injection of 1 M of ethanolamine buffer (pH 8.5) to block the residual active groups. For each sample analysis, another reference channel without the conjugated protein was also activated and blocked to eliminate non-specific binding to the surface of CM5 chip. Next, FA solution was diluted into different concentrations and injected into the analysis channel at a flow rate of 30 μL/min. The binding time of proteins and ligands was 420 s and the natural dissociation time was 420 s. All data were analyzed using Biacore T200 software.

### Statistical Analysis

All experimental data were expressed as mean ± SEM and repeated at least three times. One-way ANOVA was employed to compare the differences between multiple groups using GraphPad Prism 8 (Graph-Pad, San Diego, CA). *p* value ≤ 0.05 was considered statistically significant.

## Results

### FA Significantly Prevents Liver Fibrosis in CCl_4_-Induced Mouse Model

**FIGURE 1:**
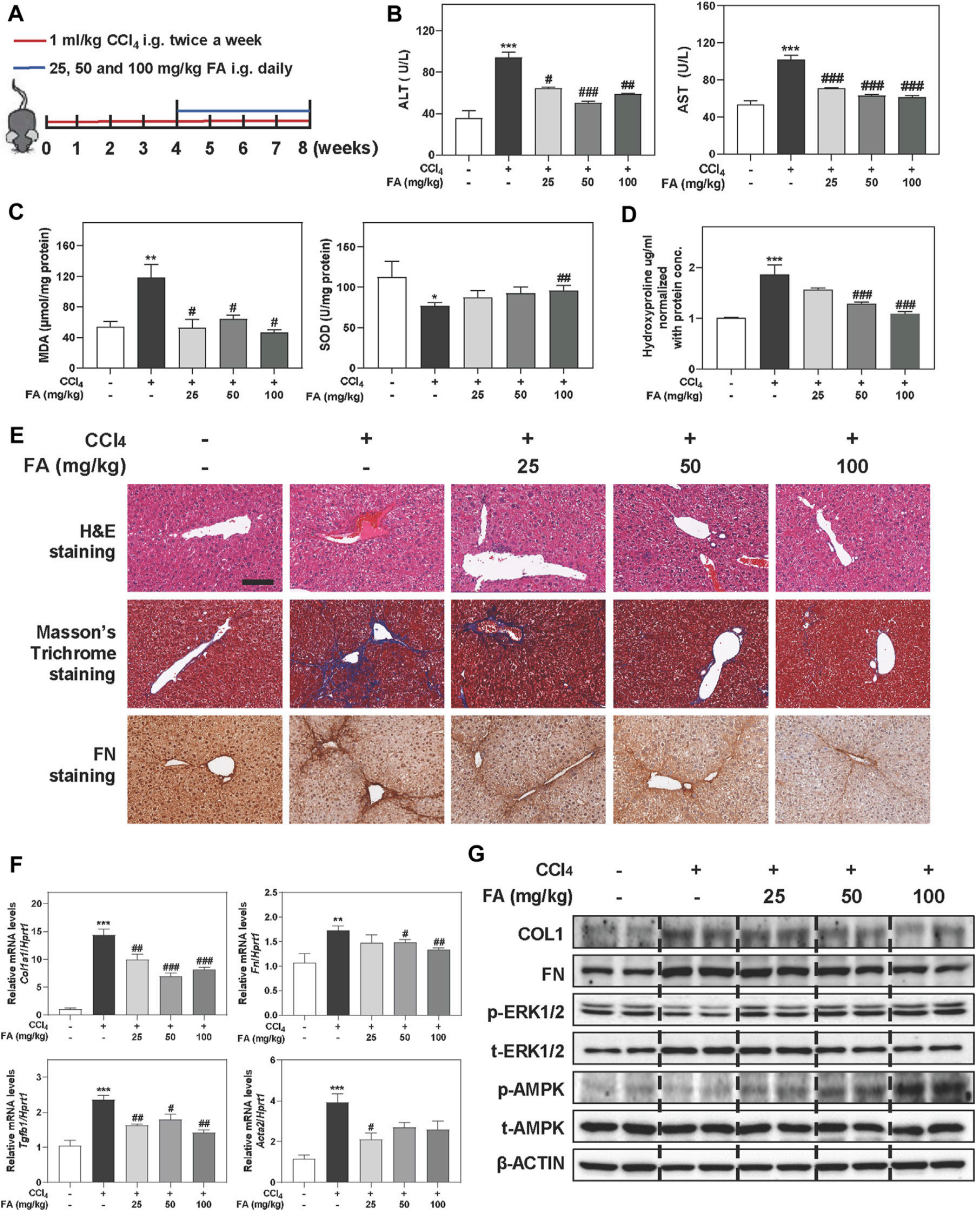
FA alleviates CCl4-induced fibrotic injury in mice. Mice were received with CCl_4_ (1 ml/kg) and different dosages (25, 50, and 100 mg/kg) of FA by gavage. **(A)** Schematic diagram of *in vivo* experiments that linked FA with anti-fibrotic therapies. **(B)** ALT and AST levels in serum. **(C)** MDA and SOD levels in livers. **(D)** Measurement of the liver hydroxyproline. **(E)** Representative images of H & E, Masson’s Trichrome and FN staining. Scale bar = 100 μm. **(F)** Relative mRNA levels of Col1a1, Fn, Tgfb1 and Acta2 were determined by qPCR and normalized using Hprt1 as an internal control. **(G)** Representative immunoblots against COL1, FN, p-ERK1/2, t-ERK1/2, p-AMPK, t-AMPK and β-ACTIN were shown. Statistical significance: *p < 0.05, **p < 0.01, ***p < 0.001, compared with control group; ^#^ *p <* 0.05, ^##^ *p* < 0.01, ^###^ *p* < 0.001, compared with CCl_4_ group. One-way ANOVA with Tukey’s post-hoc tests (*n* = 6).

To reveal the potential protective effects and detailed mechanisms of FA on liver fibrosis progression, C57BL/6J mice were first orally administered with CCl_4_ for 4 weeks to induce chronic inflammation and liver fibrosis followed by treatment with different doses of FA (25, 50 and 100 mg/kg) or vehicle control for additional 4 weeks in addition to continuous gavage of CCl_4_ (Figure 1A). The significant increased ratios of liver or spleen weight over body weight caused by CCl_4_ were slightly decreased after FA treatment (Supplementary Figure S1A). Notably, serum biochemistry assays showed that CCl_4_ significantly increased the serum levels of alanine transaminase (ALT) and aspartate transaminase (AST), which were all markedly reversed by FA administration (Figure 1B). We further determined whether FA protected liver fibrosis through the suppression of hepatic oxidative stress. As expected, the hepatic content of malondialdehyde (MDA) in CCl_4_-treated group was markedly increased than that in control group (Figure 1C, left panel), while superoxide dismutase (SOD) level was decreased in livers (Figure 1C, right panel). Interestingly, FA significantly downregulated MDA level and only high dose of FA upregulated the SOD level in livers (Figure 1C). Consistently, biochemical quantification of hepatic collagen revealed readily detectable collagen deposition in CCl_4_ treated mice but decreased when treated with FA (Figure 1D). As shown in Figure 1E and Supplementary Figure S1B, H & E and Masson’s Trichrome staining depicted that FA significantly prevented the CCl_4_-caused fibrotic responses as indicated by reduced ballooning changes of hepatocytes and area of inflammation infiltration and collagenous fibers in the liver. Immunohistochemistry staining of FN, a specific marker of liver fibrosis, indicated that FN was upregulated in CCl_4_ groups and downregulated after FA treatment. QPCR and western blot analysis further confirmed that CCl_4_-induced the upregulation of COL1 and FN were significantly inhibited by FA (Figures 1F,G and Supplementary Figure S1C). TGF-β, a principal pro-fibrotic factor, promoted the expression of Acta2 and other fibrotic indicators through small mothers against decapentaplegic (SMAD) pathways. As shown in Figure 1F, FA-induced recovery from CCl_4_ injury was accompanied with the downregulation of pro-fibrogenic mRNA encoding for Tgfb1 and Acta2. Previous studies reported that AMPK activation improved hepatic inflammation and fibrosis even after the onset of liver fibrosis associated-steatohepatitis (Zhao et al., 2020). To further investigate whether the protective effect of FA on liver fibrosis was relevant to AMPK phosphorylation, we determined the protein expression of phosphorylated AMPK and its upstream kinases, ERK1/2 and LKB1. As shown in Figure 1G and Supplementary Figure S1D, the phosphorylation of AMPK and ERK1/2, but not LKB1, was significantly increased after FA treatment compared with CCl_4_ group.

### FA Activates AMPK and Alleviates CCl_4_-Induced Oxidative Stress in Hepatocytes

**FIGURE 2:**
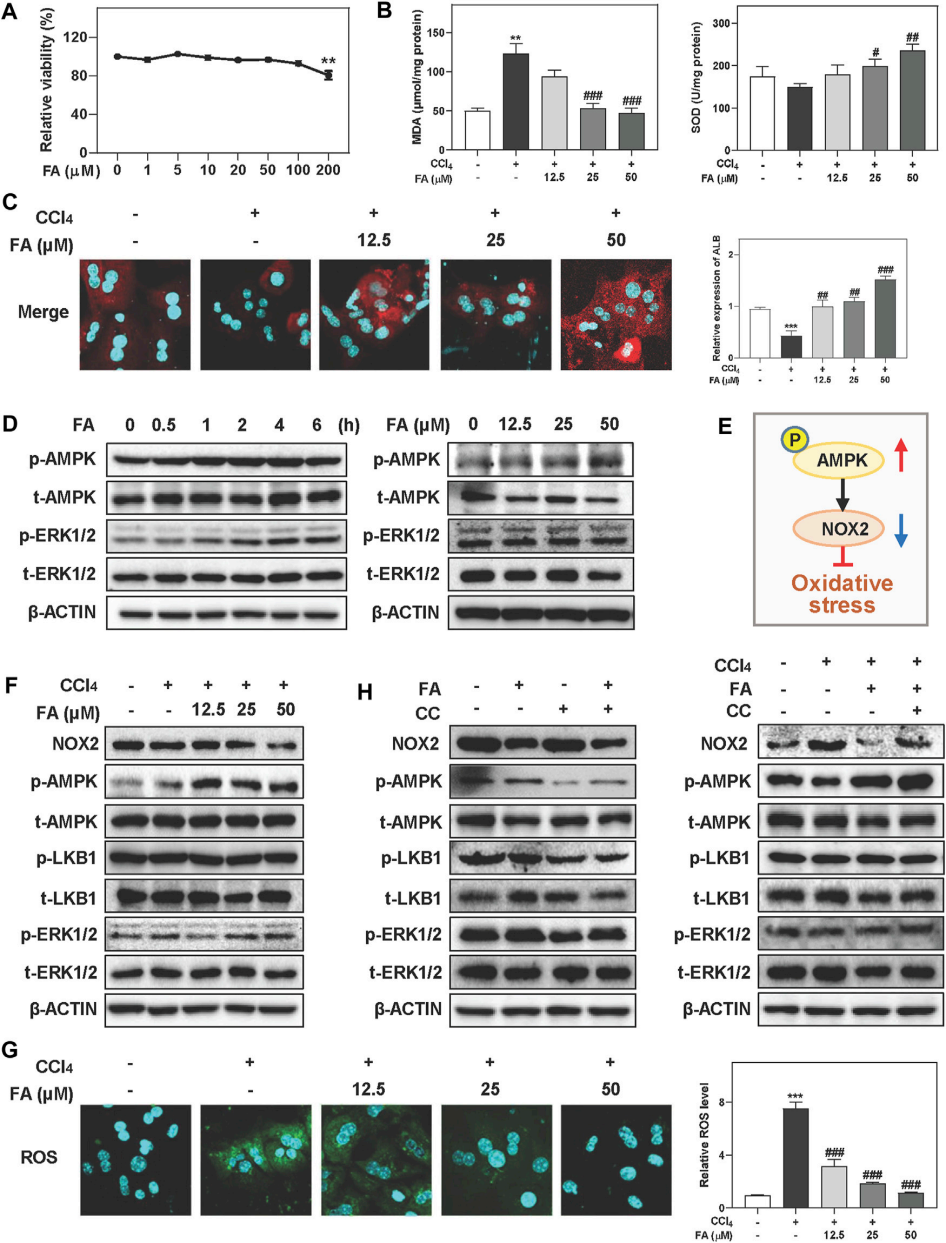
FA improves CCl4-induced oxidative stress and hepatic injury in MPHs. **(A)** A CCK-8 assay. **(B, C, F and G)** MPHs were pre-treated with FA at different concentrations for 1 h and then treated with CCl_4_ (10 mM) for another 24 h **(B)** MDA and SOD levels in MPHs. **(C)** Representative images of immunofluorescent staining of ALB in MPHs. **(D)** MPHs were treated with 25 μM FA at different time points or different concentrations of FA for 24 h. **(E)** The pathway of AMPK and NOX2 involved in oxidative stress. **(G)** Representative images of ROS immunofluorescent staining in MPHs. **(H)** After pre-treated with CC (2 μM) or FA (25 μM) or both, MPHs were administrated with CCl_4_ (10 mM) for another 2 h or 24 h **(D, F and H)** Representative immunoblots against p-AMPK, t-AMPK, p-LKB1, t-LKB1, p-ERK1/2, t-ERK1/2, NOX2 and β-ACTIN were shown. Statistical significance: **p < 0.01, ***p < 0.001, compared with control group; ^#^ *p <* 0.05, ^##^ *p* < 0.01, ^###^ *p* < 0.001, compared with CCl_4_ group. One-way ANOVA with Tukey’s post-hoc tests (*n* = 3).

It is well known that oxidative stress is particularly relevant to liver fibrosis progression. Given the hepatoprotective and anti-fibrotic effects of FA *in vivo* experiments, we next examined whether FA attenuated oxidative hepatic injury in MPHs treated with CCl_4_. First, CCK-8 assay showed that FA below 100 μM didn’t cause any cytotoxicity in MPHs (Figure 2A). Therefore, 12.5, 25 and 50 µM of FA were used for dose-dependent experiments and 25 µM of FA was used for following *in vitro* assays. As shown in Figure 2B, consistent with the results *in vivo*, CCl_4_ treatment markedly increased the hepatic level of MDA, which was significantly inhibited by FA at medium and high doses. In addition, 25 and 50 μM FA were able to significantly reverse the decrease of SOD caused by CCl_4_ administration. Immunofluorescent images further showed that the expression of ALB, a marker of hepatocyte function, had a significant increase in FA treated groups compared with CCl_4_ group (Figure 2C). Of note, FA significantly induced the activation of AMPK and ERK1/2 after 2 h treatment and peaked at 4 h (Figure 2D, left panel and Supplementary Figure S2A). Additionally, medium and high doses of FA rapidly and significantly induced AMPK phosphorylation at 2 h but didn’t affect the expressions of ERK1/2 and LKB1 (Figure 2D, right panel and Supplementary Figures S2B,C). Considering that AMPK activation attenuated NOX2-induced excessive ROS generation and oxidative stress (Figure 2E) (Rodriguez et al., 2020), we measured the NOX2 expression after FA and CCl_4_ treatment with or without the presence of AMPK inhibitor, CC. As shown in Figure 2F and Supplementary Figure S2D, the protein levels of NOX2 were significantly decreased after FA treatment compared with CCl_4_ group. Although co-treatment of FA and CCl_4_ barely affected the phosphorylation of LKB1 and ERK1/2 compared with CCl_4_ treatment alone, FA significantly increased the phosphorylation of AMPK. In addition, FA treatment significantly decreased the ROS production in MPHs caused by CCl_4_ (Figure 2G). Next, we examined whether the inhibition of AMPK activation had any influence on FA-induced hepatoprotective effects. In agreement with our hypothesis, pretreatment of MPHs with CC (2 μM) not only completely inhibited FA-induced AMPK activation but also reversed FA-induced downregulation of NOX2, while the levels of p-ERK1/2 and p-LKB1 were not changed (Figure 2H, left panel and Supplementary Figure S2E). Interestingly, the level of NOX2 was significantly decreased and the levels of p-AMPK was increased after FA treatment compared with CCl_4_-treated group, which was blocked by CC administration. Again, no significant change of ERK1/2 and LKB1 expression was observed (Figure 2H, right panel and Supplementary Figure S2F).

### FA Activates AMPK and Relieves LPS-Induced Inflammation in Macrophages

**FIGURE 3:**
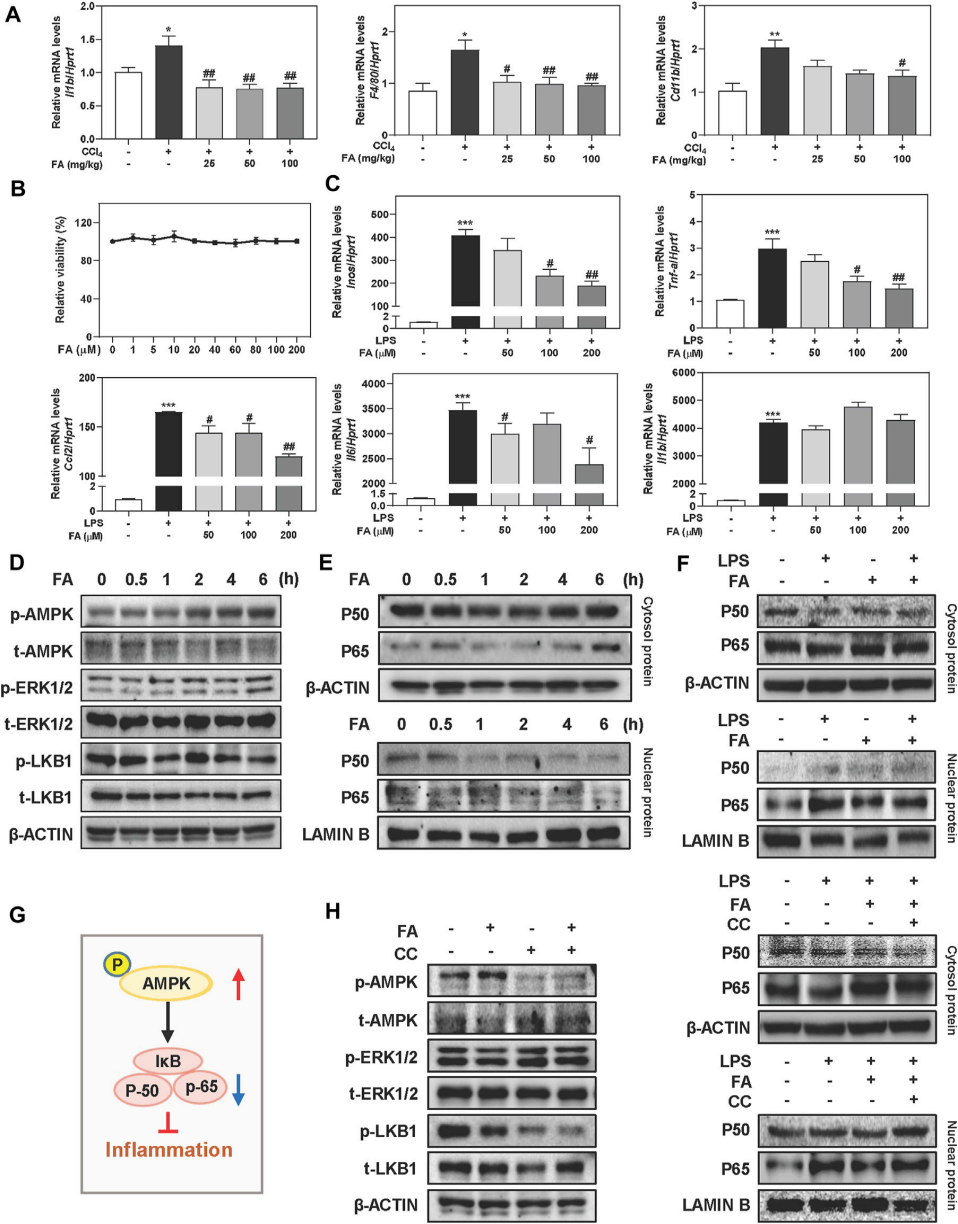
FA suppresses LPS-induced macrophage activation in RAW cells. **(A)** Relative hepatic mRNA levels of Il1b, F4/80 and Cd11b were determined by qPCR and normalized using Hprt1 as an internal control in mice. **(B)** A CCK-8 assay. **(C)** RAW cells were pre-treated with FA at different concentrations for 1 h and then treated with LPS (100 ng/ml) for another 4 h. **(D, E)** RAW cells were treated with 100 μM FA at different time points. **(F, H)** After treated with CC (10 μM) or FA (100 μM) or both, RAW cells were administrated with LPS for another 4 h. **(G)** The pathway of AMPK and NF-κB involved in inflammation. **(D-F and H)** Representative immunoblots against p-AMPK, t-AMPK, p-ERK1/2, t-ERK1/2, p-LKB1, t-LKB1, P50, P65, β-ACTIN and LAMIN B were shown. Statistical significance: *p < 0.05, ***p < 0.01, ***p < 0.001, compared with control group; ^#^ *p <* 0.05, ^##^ *p* < 0.01, compared with LPS group. One-way ANOVA with Tukey’s post-hoc tests (*n* = 3).

In addition to oxidative stress response, another key driver responsible for liver fibrosis is macrophage activation-associated inflammatory response, which promotes us to investigate the anti-inflammatory effects of FA both *in vivo* and *in vitro*. As shown in Figure 3A, hepatic mRNA expressions of interleukin-1 beta (Il1b) and macrophage markers including F4/80 and Cd11b were significantly increased after CCl4 treatment compared to control group, which were reversed by FA treatment. Furthermore, pro-inflammatory gene transcript levels of chemokine (C-C motif) ligand 2 (Ccl2) and tumor necrosis factor alpha (Tnfa) in the livers of CCl4-treated mice were markedly reduced post FA administration (Supplementary Figure S3A). Based on the observations *in vivo*, we next sought to determine the *in vitro* consequence of FA on RAW cells induced by LPS. Results showed that FA didn’t affect cell viability up to 200 μΜ (Figure 3B). After LPS intervention for 4 h, several inflammatory-related genes expression were significantly upregulated, such as inducible nitric oxide synthase (Inos), Tnf-a, Ccl2, and Il6, which were downregulated by FA treatment except for Il1b (Figure 3C). As depicted in Figure 3D and Supplementary Figures S3B,C, administration of FA significantly induced the phosphorylation of AMPK as well as p-ERK1/2 after 2 h treatment and peaked at 6 h, but didn’t affect or even slightly decrease the expression of p-LKB1. The activation of NF-κB was crucial in the process of hepatic inflammation by translocating its subunits (P50 and P65) from cytoplasm to nucleus and promoting the release of cytokines (Catrysse and van Loo, 2017). As expected, FA promoted the translocation of P50 and P65 from nucleus to cytoplasm in a time-dependent manner (Figure 3E and Supplementary Figure S3D). In addition, FA was able to markedly decrease the LPS-induced nuclear translocation of P50 and P65 (Figure 3F and Supplementary Figure S3E **)**. We further determined if FA-induced activation of AMPK was correlated to the inhibition of NF-κB nuclear translocation (Figure 3G). As shown in Figure 3H, left pane**l** and Supplementary Figure S3F, FA-induced AMPK phosphorylation was completely blocked by CC (10 μM). Co-treatment with FA and CC significantly decreased the phosphorylation of LKB1 but not affect ERK1/2 level. Furthermore, with the presence of CC, FA was unable to prevent the nuclear translocation caused by LPS (Figure 3H, right panel and Supplementary Figure S3G). These findings indicated that FA inhibited inflammatory responses through the activation of AMPK and subsequent inhibition of NF-κB in macrophages.

### FA Activates AMPK and Alleviates TGF-β-Induced Fibrotic Response in HSCs

**FIGURE 4:**
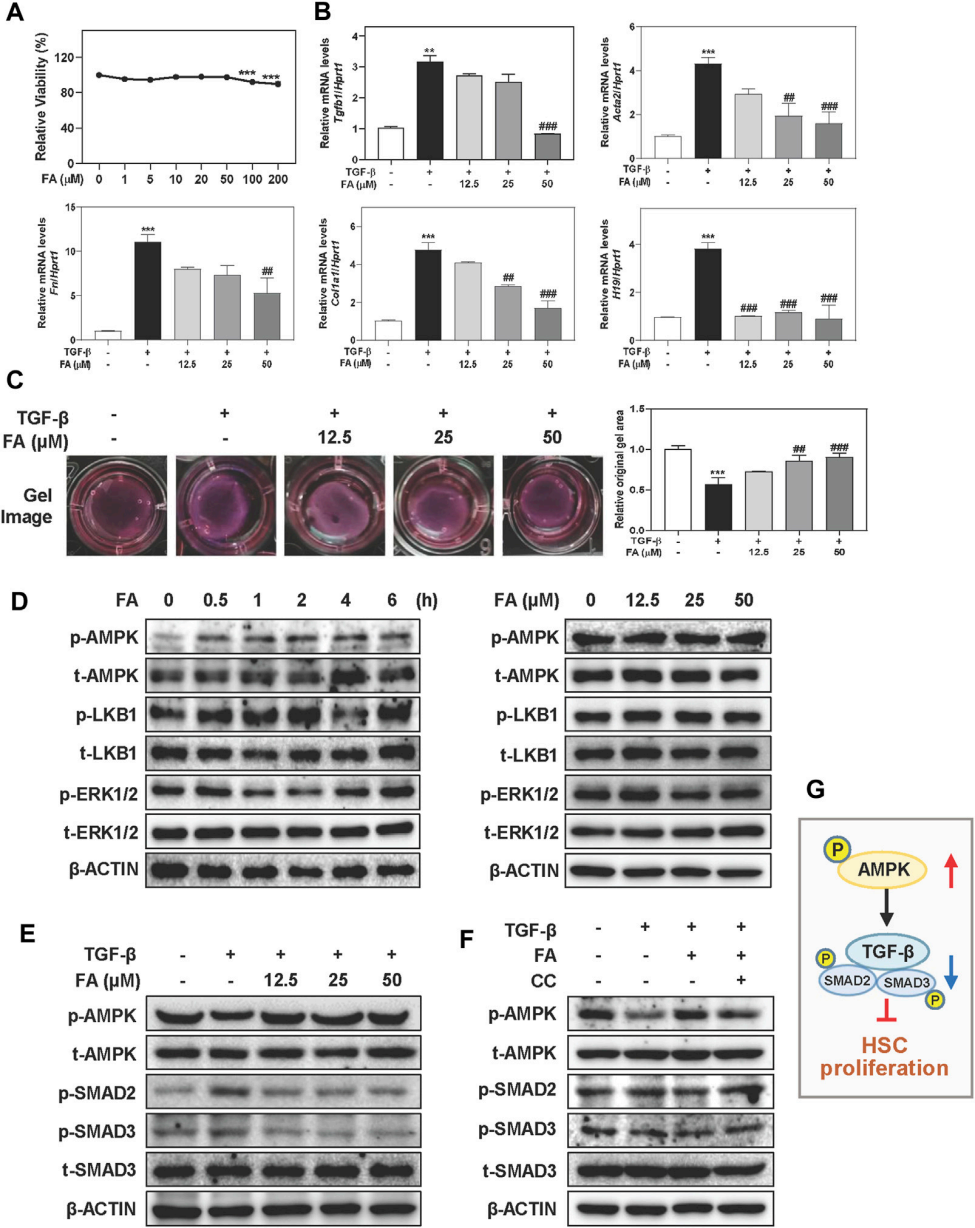
FA attenuates TGF-β-induced HSC activation and fibrotic responses in LX-2 cells. **(A)** A CCK-8 assay. After pre-treated with FA at different concentrations for 1 h, LX-2 cells were treated with TGF-β (5 ng/ml) for another 24 h **(B and C)** or 6 h **(E)**. **(B)** Relative mRNA levels of Tgfb1, Acta2, Fn, Col1a1 and H19 were determined by qPCR and normalized using *Hprt1* as an internal control. **(C)** Representative collagen gel images from three independent experiments were shown. **(D)** LX-2 cells were treated with 25 μM FA at different time points or different concentrations of FA for 24 h. **(F)** After treated with CC (2 μM) or FA (25 μM) or both, LX-2 cells were administrated with TGF-β for another 6 h **(D-F)** Representative immunoblots against p-AMPK, t-AMPK, p-LKB1, t-LKB1, p-SMAD2, p-SMAD3, t-SMAD3, p-ERK1/2, t-ERK1/2 and β-ACTIN were shown. **(G)** The pathway of AMPK, TGF-β and SMADs involved in HSC proliferation. Statistical significance: **p < 0.01, ***p < 0.001, compared with control group; ^##^ *p* < 0.01, ^###^ *p* < 0.001, compared with TGF-β group. One-way ANOVA with Tukey’s post-hoc tests (*n* = 3).

In order to explore whether FA prevented the activation of HSCs, main ECM-producing cells in livers, through the phosphorylation of AMPK, we incubated LX-2 cells, a human HSC cell line, with TGF-β and increasing concentrations of FA *in vitro*. As shown in Figure 4A, we conducted CCK-8 assay and selected 12.5, 25 and 50 μM of FA for dose-dependent experiments and 25 μM of FA for other *in vitro* studies. 50 μM of FA significantly downregulated the mRNA levels of genes associated with HSC activation like Tgfb1 and Fn compared to TGF-β group. In addition, the mRNA levels of Acta2 and Col1a1 were dramatically increased in TGF-β-treated group, which were significantly reduced after FA treatment at medium and high doses (Figure 4B). We recently reported that the aberrant expression of long noncoding RNA (lncRNA) H19 was responsible for HSC activation and liver fibrosis progression in multiple mouse models (Wang et al., 2021). As shown in Figure 4B, TGF-β-induced upregulation of H19 was significantly reversed by FA in LX-2 cells. According to the results of gel contraction analysis, the relative original gel area in TGF-β treated group was significantly reduced due to a strong contraction force compared to control group, which was markedly reversed by FA treatment at 25 and 50 μM (Figure 4C). Meanwhile, the effects of FA alone were not statistically significant (Supplementary Figure S4A). As shown in Figure 4D, left panel and Supplementary Figure S4B, although FA did not affect LKB1 expression, it caused a time-dependent increase of p-AMPK phosphorylation at 0.5 h and peaked at 2 h in LX-2 cells. Interestingly, with the presence of FA, p-ERK1/2 was slightly decreased from 1 to 2 h while was continually increased after 4 h. Consistent with the results above, FA also markedly activated the phosphorylation of AMPK without affecting the levels of p-LKB1 and p-ERK1/2 in LX-2 cells (Figure 4D, right panel and Supplementary Figure S4C). Given the strong influence of FA on AMPK activation, we further explored whether FA prevented HSC activation by regulating AMPK and its related SMAD2/3 signaling. As shown in Figure 4E and Supplementary Figure S4D, FA reversed the downregulation of phosphorylated AMPK and suppressed the phosphorylation of p-SMAD2 and p-SMAD3 caused by TGF-β administration. Notably, CC significantly inhibited FA-induced upregulation of AMPK, accompanied by a robust increase of p-SMAD2 and p-SMAD3 except for LKB1 (Figure 4F and Supplementary Figures S4E,F). These results confirmed that the anti-fibrotic effects of FA depended on AMPK activation and its downstream SMAD2/3 pathways (Figure 4G).

### PTP1B is Required for FA-Mediated Phosphorylation of AMPK

**FIGURE 5:**
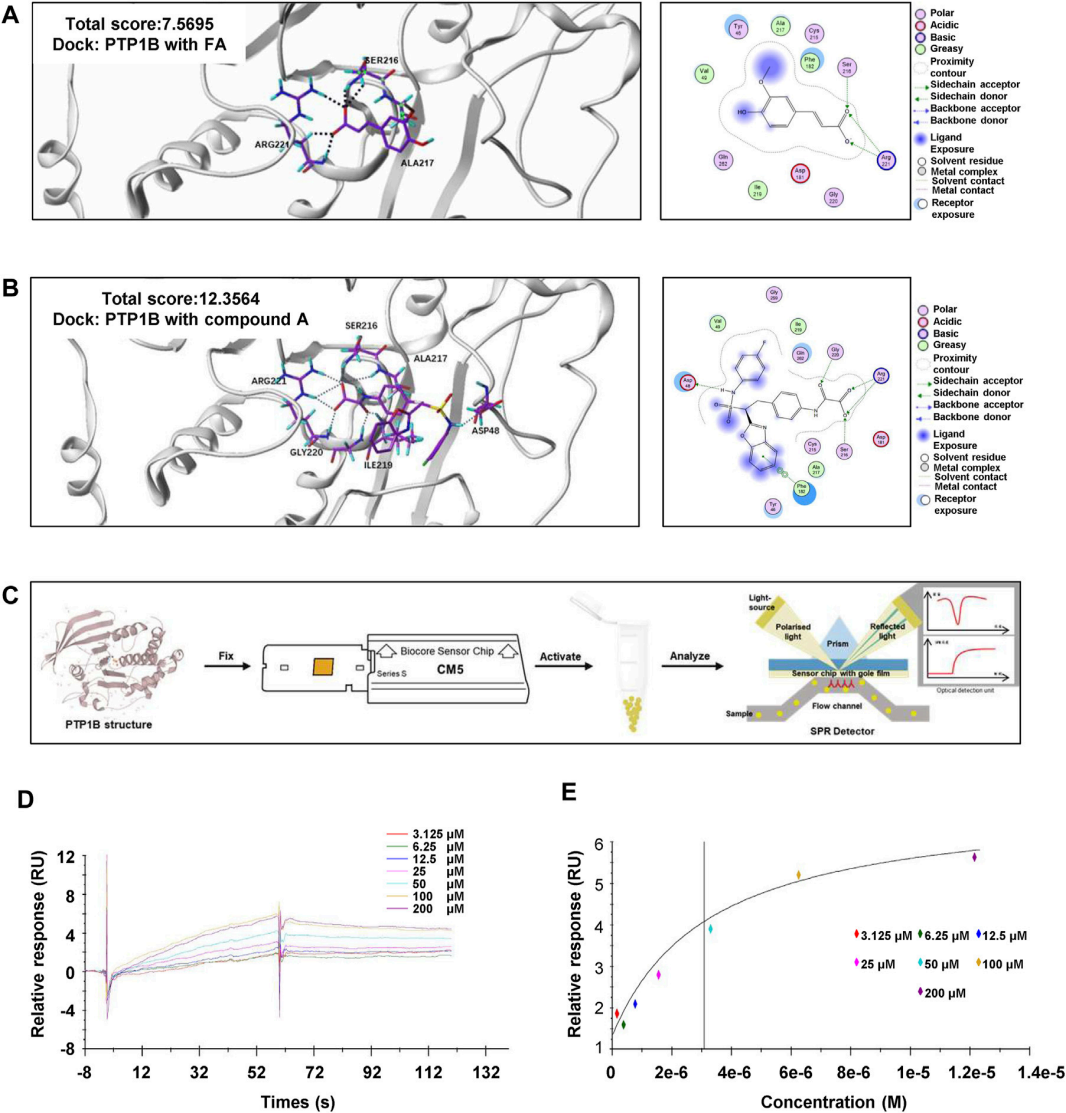
FA directly binds to PTP1B. **(A)** Representative images for the binding mode of FA and PTP1B with the crystal structure of PTP1B (PDB ID: Ser216, Arg221, Ala217). **(B)** Representative images for the binding mode of compound A and PTP1B with the crystal structure of PTP1B (PDB ID: Arg221, Ser216, Ala217, Gly220, Ile219 and Asp48). **(C)** The process flow diagram of SPR. **(D)** The concentration gradient binding curves of protein PTP1B with FA (3.125, 6.25, 12.5, 25, 50, 100 and 200 μM). **(E)** The representative binding curve of FA binding to PTP1B. All the experiments were conducted three independent times.

Based on the reported above, we demonstrated that FA was able to upregulate the phosphorylation of AMPK in different liver cells without affecting LKB1 phosphorylation. Given that PTP1B deficiency contributed to oxidative stress and hepatic lipid disorders, we also examined the mRNA and protein levels of PTP1B after FA treatment. Interestingly, FA had no effect on PTP1B expression in hepatocytes, macrophages as well as HSCs, with or without the presence of different model drugs (Supplementary Figures S5A–E). Lines of evidence indicated that PTP1B dephosphorylated multiple protein kinases and caused the modulation of multiple signaling pathways (Niu et al., 2020). Given that the regulatory effects of PTP1B on AMPK activation were not only affected by its expression but also relied on its phosphatase activity, FA might mediate AMPK phosphorylation through the regulation of PTP1B activity. To test this hypothesis, we first examined whether FA directly bound to PTP1B using molecular docking and SPR assays. Molecular docking was used to construct a virtual FA-PTP1B structure model. As shown in Figures 5A,B, the receptor and ligand of PTP1B were prepared and the bonding mode was predicted by the Surflex-Dock program. The total score of PTP1B towards FA was 7.5695 and towards its inhibitor, compound A, was 12.3564. Correspondingly, PTP1B interacted with FA by forming the hydrogen bonds with catalytically active sites, Arg221, Ser216 and Ala217, and interacted with compound A by forming the hydrogen bonds with Arg221, Ser216, Ala217, Gly220, Ile219 and Asp48. Comparing the binding sites of PTP1B with FA or compound A, we pointed out that Arg221 might become the most critical residue responsible for the interaction between PTP1B and FA. Consistent with our hypothesis, FA might have a moderate binding affinity with PTP1B and act as a potential antagonist of PTP1B. Additionally, SPR is one of the most prominent optical biosensor technologies and was used to gain further insight into the binding affinity of PTP1B to FA (Figure 5C). As expected, binding of FA to PTP1B was dose-dependent, exhibiting a association-dissociation process (Figure 5D). The response units at equilibrium were plotted against FA concentrations and the dissociation constant (KD) was calculated by non-linear regression. As shown in Figure 5E, PTP1B directly bound to FA with a KD value of 3.474 μM.

### The Chemical Structure of FA is Crucial for AMPK Phosphorylation

**FIGURE 6:**
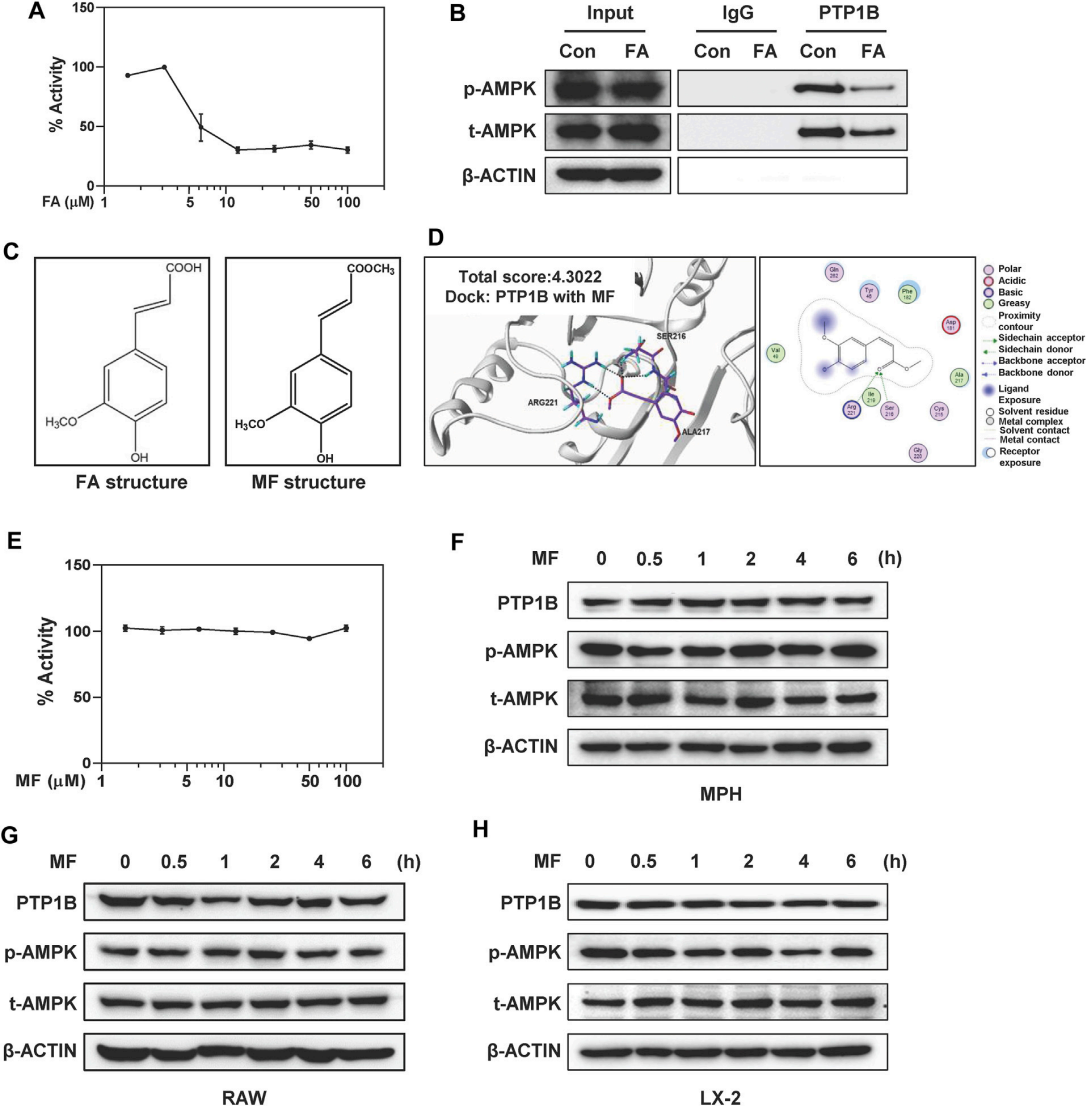
FA directly inhibits PTP1B activity but MF doesn’t affect PTP1B and AMPK signalings in different liver cells. **(A)** Inhibition of the catalytic activity of PTP1B by FA was shown. **(B)** LX-2 cells were treated with 25 μM FA for 24 h. Immunoprecipitation using monoclonal anti-PTP1B or IgG, followed by western blot analysis using rabbit polyclonal anti-p-AMPK and t-AMPK. 10% input was prepared as a positive control and ß-ACTIN was used as a loading control. **(C)** Chemical structures of FA and MF. **(D)** Representative images for the binding mode of MF and PTP1B. **(E)** Inhibition of the catalytic activity of PTP1B by MF was shown. **(F and H)** MPHs, LX-2 cells and **(G)** RAW cells were treated with 25 or 100 μM MF at different time points. **(F-H)** Representative immunoblots against p-AMPK, t-AMPK, PTP1B and ß-ACTIN were shown. One-way ANOVA with Tukey’s post-hoc tests (*n* = 3).

Guided by the results that FA had a strong binding ability with PTP1B (Figure 5) but not AMPK (Supplementary Figure S5F), we further examined whether FA regulated AMPK phosphorylation by affecting the enzyme activity of PTP1B. As shown in Figure 6A, FA rapidly inhibited the activity of PTP1B more than 50% in a dose-dependent manner, suggesting that it might behave as a potential inhibitor of PTP1B. In addition, co-immunoprecipitation assay was employed to test whether and how FA regulated the interaction between PTP1B and AMPK. In support of our hypothesis, the interaction between PTP1B and AMPK was weakened after FA treatment, possibly because FA competitively occupied the catalytic domain of PTP1B, which was also the binding site for phosphorylated AMPK (Figure 6B).

Combined with the above results, we further speculated that the inhibitory effect of FA on PTP1B might be related to the predicted binding mode, especially the formation of hydrogen bond between -COOH on FA and Arg221 and Ser216 of PTP1B. MF is a methyl ester of FA (Cheng et al., 2019). The only difference between FA and MF was a -COOH group in the side chain of FA while a -COOCH3 in the same position of MF (Figure 6C). Interestingly, MF had a low predicted binding score and the critical hydrogen bond between -OH with Arg221 was missing (Figure 6D). In supporting of molecular docking results, MF had minimal effects on the enzymatic activities of PTP1B at as high as 100 μM (Figure 6E). Furthermore, time- and dose-course analyses by western blot revealed that MF didn’t affect the phosphorylation of AMPK as well as the expression of PTP1B in MPHs, RAW cells and LX-2 cells, respectively (Figures 6F–H and Supplementary Figures S6A–F).

## Discussion

**FIGURE 7:**
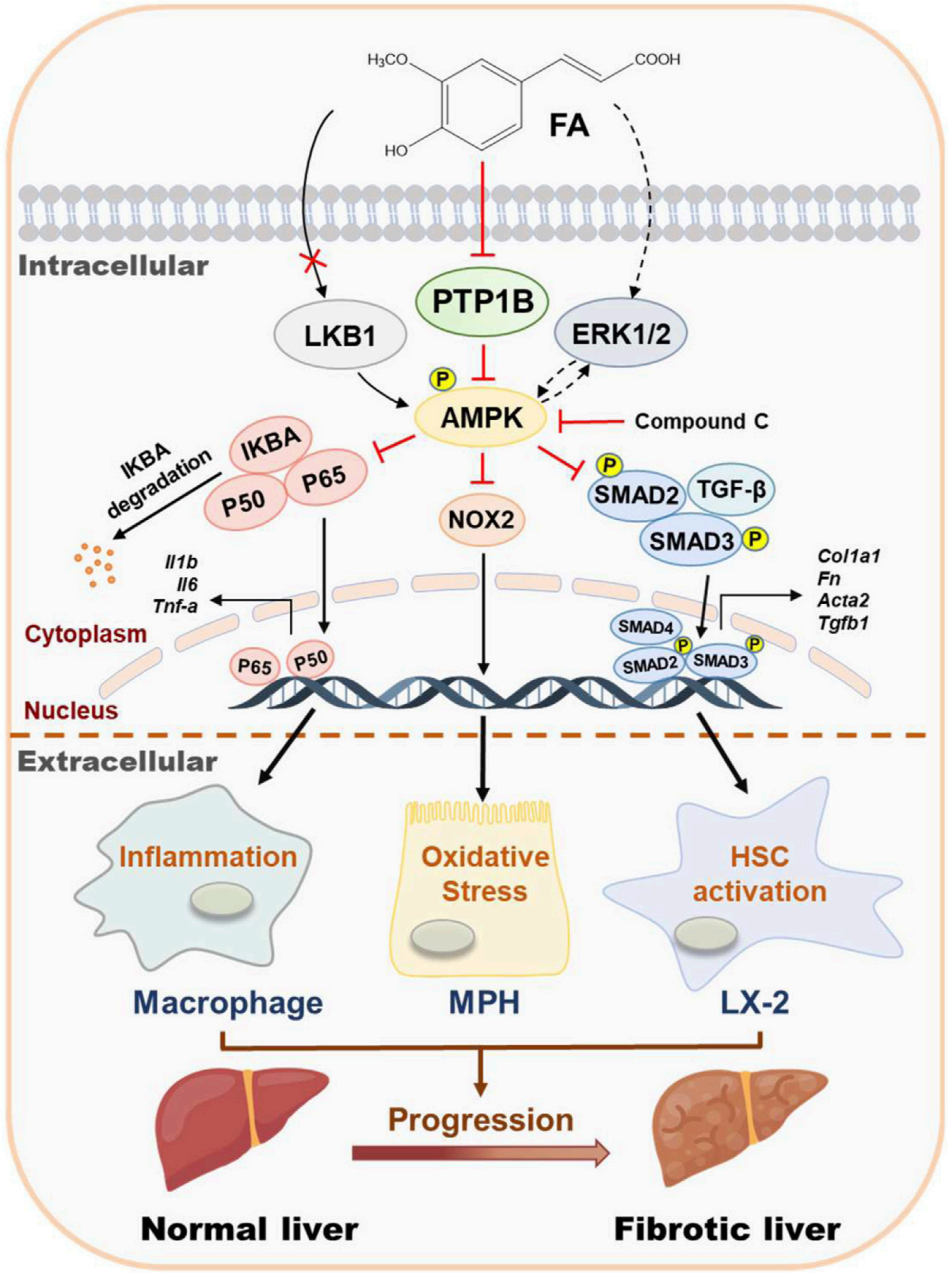
Schematic diagram of the proposed mechanisms underlying the protective effects of FA on oxidative stress, inflammation in macrophages and HSC activation during liver fibrosis. FA ameliorated CCl_4_-induced oxidative stress in hepatocytes, relieved LPS-induced inflammation in macrophages and alleviated TGF-β-induced fibrotic response in HSCs. The potential mechanism was that FA directly bound to and inhibited the activity of PTP1B, eventually leading to the phosphorylation of AMPK.

Liver fibrosis results from multiple pathogenic factors such as oxidative stress, persistent inflammation and lipid disorders and is characterized by the net accumulation of ECM, leading to a serious threat to human health. Indeed, although several anti-fibrotic drugs are in development, high research and development costs and the risk of side effects restrict their clinical applications. In the past decades, natural products are reported to possess novel biological activities, thus making them a rich source of lead compounds for new drug discovery for the treatment of chronic liver diseases. FA, one of the natural representative phenolic acids, has provided opportunities for the treatment of acute or chronic diseases. FA exerts a broad spectrum of biological activities, including ameliorating inflammation, preventing microbial invasion and alleviating lipid droplets deposition, which may be related to antioxidant or antifibrosis but systemic pharmacological evaluation and clear underlying mechanism are still missing. The current study reported that FA markedly protected against pathological changes and liver fibrosis caused by CCl_4_ in mice. It also inhibited hepatic oxidative stress, inflammation in macrophages and HSC activation at the cellular level. Mechanically, our data suggested that the beneficial effects of FA were due to its activation of AMPK by directly binding to PTP1B and further inhibiting PTP1B activity (Figure 7). Therefore, a better understanding of the protective mechanisms of FA may yield important insights into the treatment of liver fibrosis and advanced complications.

AMPK, as a pivotal player in maintaining cellular energy homeostasis, is reported to regulate multiple metabolic processes. Previous articles indicated that the loss of AMPK activation significantly aggravated liver inflammation and lipid disorders in mouse fibrotic models (Lin et al., 2020; Zhao et al., 2020). In addition, the protective functions of AMPK phosphorylation are also highlighted recently, including preventing oxidative stress and attenuating alcoholic liver disease progression (Lu et al., 2021). Although AMPK activation is also documented to suppress the expression of bile acid transporters in hepatocytes and further be responsible for estrogen-induced cholestasis, which may result from the sustained over-activation of AMPK (Li et al., 2017), loss of AMPK promotes steatosis development in mice without affecting normal physiological functions (Boudaba et al., 2018; Zhao et al., 2020). Thus, AMPK activation may serve as a reversal point of fibrotic liver damage. If so, pharmaceutical interventions that activate AMPK in livers may represent potential approaches to treat hepatic fibrosis. Here, we reported that FA strikingly protected against oxidative stress and fibrotic responses both *in vivo* and *in vitro* by facilitating AMPK phosphorylation (Figures 1–4).

CCl_4_ is a multicellular injury model and the consequences it induced are similar to the clinical pathological phenotype of liver fibrosis. Hepatocytes, which account for 60% of the total liver cells and 80% of the liver volume, are the primary source of sensing “danger signals” in liver fibrosis. Under pathological processes, damaged hepatocytes release damage-associated molecular patterns (DAMPs), stimulate the liver-resident macrophages to release pro-inflammatory cytokines/chemokines and result in the recruitment of macrophages that promote HSC survival and activation (Cha et al., 2018; Hirsova et al., 2016). Activated AMPK in hepatocytes not only suppressed the apoptosis of hepatocytes and NOX2-derived ROS production (Rodriguez et al., 2020) but also inhibited the production of endothelial pro-inflammatory cytokines (Hawley et al., 2016). The current work provided evidence that FA exerted excellent anti-oxidant effects and protected hepatocyte injury through AMPK activation and subsequent NOX2 inhibition (Figure 2), indicating that the protective effects of FA against oxidative stress on hepatocytes might further reduce the release of cytokines/chemokines and macrophage activation. On the other side, sensitized hepatocytes respond to the release of inflammatory cytokines and further result in hepatic damage and progression of hepatocyte steatosis (Nagy et al., 2016; Zhang et al., 2019). Meanwhile, the activation of AMPK is reported to block NF-κB translocation from the cytosol to the nucleus in activated macrophages (Zhai et al., 2018). Except for the role of FA alone in preventing the nuclear translocation and activation of NF-κB, we further reported that FA reduced the release of inflammatory cytokines in LPS-stimulated RAW cells through the inhibition of NF-κB translocation (Figure 3). In addition, AMPK activation was reported to antagonize TGF-β-induced SMAD3 activation and fibrogenic response in HSCs by directly targeting transcriptional coactivator p300, and finally, improve hepatocellular dysfunction in the liver (Lim et al., 2012). Our results further suggested that FA directly activated AMPK phosphorylation and inhibited HSC activation through TGF-β/SMADs pathway (Figure 4). These results indicated that FA protected hepatocytes from the “second hit” by suppressing inflammatory reactions in macrophages and preventing HSC activation. Furthermore, CC completely inhibited AMPK activation and abrogated FA-induced hepatoprotective effects in different liver cells, suggesting an indispensable role of AMPK activation underlying the therapeutic effects of FA.

Our results indicate that the FA-induced activation of AMPK is not related to LKB1 but regulated by an alternative mechanism. Similar to AMPK, emerging proofs suggest that PTP1B participates in the course of metabolic and fibrotic liver diseases (Zhang et al., 2015). Deficiency of PTP1B is also reported to effectively restore the dampened phosphorylation of AMPK and hyperactivate phosphorylation of mTOR and Raptor in mice fed with HFD (Kandadi et al., 2015; Xu et al., 2019). Considering the low expressions of NF-κB and NOX2 and inhibited inflammation in liver-specific PTP1B knockout mice (Hsu et al., 2020), we speculated whether these changes are related to AMPK activation in livers and our hypothesis remains to be identified in the future. In the current study, we found that FA relived hepatic fibrosis by, at least partly, directly binding to and acting as a PTP1B antagonist, which further confirmed our hypothesis that the binding ability between FA and AMPK was weaker than that between FA with PTP1B (Figure 5). In support of molecular docking and SPR studies, we further showed that although no significant change of PTP1B expression was detected, its enzyme activity was significantly inhibited after FA administration (Figure 6). Collectively, our study provided critical evidence that FA alleviated fibrotic liver injury through AMPK phosphorylation, which was relied on the inhibition of PTP1B activity.

There are several critical amino acid residues (Arg221, Ala217, Asp 48, Asp181, Gly183, Gly220, Lys116, Phe182 and Tyr46) of PTP1B were found to directly interact with potential inhibitors isolated from natural products (Jung et al., 2017; Sharma et al., 2020; Hu et al., 2021). Notably, Arg221 exerts an important function in optimizing salt bridge interactions with the phosphate bound to the catalytic site and stabilizing the phosphoryl-enzyme intermediator (Niu et al., 2020). Here we reported that Arg221 might play a vital role in the inhibition of PTP1B by FA. Our molecular docking results revealed that the MF did not fully occupy the positions of Arg221 and might lead to a low binding ability of MF to PTP1B. In order to explore whether the differences in PTP1B inhibition might be related to the structural differences, we compared PTP1B activities after FA and MF treatments, respectively. Although MF was able to attenuate HSC activation and liver fibrosis by inhibiting TGF-β1/SMADs and NOX4/ROS signaling pathways (Cheng et al., 2019), there was no alteration of PTP1B activity observed after MF administration. Additionally, one of the unavoidable ways to generate FA is through the ester hydrolyzation of MF (Mathew and Abraham, 2004). Interestingly, MF took a longer time than FA to slightly increase the phosphorylation of AMPK (Figure 6). This phenomenon may be because MF is able to be metabolized into FA and subsequently inhibit PTP1B activity to activate AMPK. These results encourage that FA and its derivatives with similar structures may provide a possible opportunity for the development of potential PTP1B inhibitors to treat fibrotic liver injury.

In conclusion, our findings indicated that FA exhibited therapeutic effects against fibrotic liver injury both *in vivo* and *in vitro* by effectively inhibiting hepatic oxidative stress, inflammation and HSC activation. Importantly, we clarified that the anti-fibrotic effect of FA was primarily attributed to the inhibition of PTP1B activity and subsequent AMPK phosphorylation. Collectively, as illustrated in Figure 7, the hepatoprotective effects of FA and underlying complicated mechanisms by which FA activates AMPK are emphasized and vital evidence inspiring the development of FA-based innovative drug candidates for the treatment of liver fibrosis and related complications is provided.

## Data Availability

The original contributions presented in the study are included in the article/Supplementary Material, further inquiries can be directed to the corresponding author.

## Abbreviations

ALT, alanine aminotransferase; AST, aspartate aminotransferase; CC, compound C; CCl4, carbon tetrachloride; COL1, collagen 1; FN, fibronectin; TGF-β, transforming growth factor-beta; LPS, lipopolysaccharide; MPH, mouse primary hepatocyte; FA, ferulic acid; MF, methyl ferulate; PTP1B, protein tyrosine phosphatase 1B; AMPK, adenosine monophosphate-activated protein kinase; LKB1, liver kinase B1; ERK1/2, extracellular-signal-regulated protein kinase 1/2; HSC, hepatic stellate cell; ECM, extracellular matrix; SPR, surface plasmon resonance; α-SMA, alpha-smooth muscle actin; NOX2, nicotinamide adenine dinucleotide phosphate oxidase-2; NF-κB, nuclear factor kappa B; SMAD, small mothers against decapentaplegic.
