# Supplementary material for: Ferulic Acid Ameliorates Hepatic Inflammation and Fibrotic Liver Injury by Inhibiting PTP1B Activity and Subsequent Promoting AMPK Phosphorylation
Source: Front Pharmacol. 2021 Sep 8;12:754976. doi: 10.3389/fphar.2021.754976 (PMC8455917; doi:10.3389/fphar.2021.754976)
Supplement: Supplementary file 1 [file DataSheet1.docx]

**Supplementary material**

**1. Supplementary materials and methods**

**1.1 Cell viability assay**

The cytotoxic effects of FA on MPHs, LX-2 cells and RAW 264.7 cells were tested using a CCK-8 assay kit (C6005) from New Cell & Molecular Biotech Co., Ltd (Suzhou, China) according to the manufacturer’s instructions. Cells were seeded in 96-well plates at different density (MPH: 1 × 10^4^/well; LX-2: 1 × 10^4^/well; RAW cells: 2 × 10^4^/well) and cultured for 24 h. Then, cells were treated with FA at different concentrations for 24 h. At the end of treatment, CCK-8 reagent was added to each well for another 2-h incubation. The absorbance was measured at 450-nm wavelength by xMark™ Microplate Absorbance Spectrophotometer (Bio-Rad, Hercules, USA).

**1.2** **Measurement of the liver functional enzyme activities, malondialdehyde and hydroxyproline**

Mice were sacrificed at the end of the treatment to obtain serum for analysis. Serum levels of ALT and AST were inspected using assay kits from Sigma (St. Louis, USA) following the manufacturer’s instructions. Liver samples were lysed and homogenized in RIPA buffer for analysis. Liver hydroxyproline, liver and cellular SOD and MDA were assessed using commercially hydroxyproline kits (#A030-1-1), SOD assay kits (#A001-3-2) and MDA assay kits (#A003-1-2) from Jiancheng Bioengineering Institute (Nanjing, China) according to manufacturer’s instruction, respectively.

**1.3 Collagen gel contraction assay**

Cells were premixed with 500 µl of 1 mg/ml collagen gels (354236) from BD Biosciences (San Jose, USA) and subsequent seeded in a 24-well dish in triplicate for each group before equilibrated at 37 °C with 30 min. After solidified and separated from the walls of 24-well dish, collagen gels with cells were incubated with 10 % FBS/DMEM medium containing different drugs for 3 d. Image J was used to measure the area of collagen gel.

**1.4 Western Blot analysis**

Total proteins were isolated from the liver tissues of mice and different cells using RIPA buffer from Beyotime Institute of Biotechnology (Shanghai, China). Nuclear and cytoplasmic proteins were extracted by nuclear and cytoplasmic protein extraction kit from KeyGEN BioTECH (Nanjing, China). Equivalent protein was resolved on 10 % SDS-PAGE gel, transferred to PVDF membranes (Merck Millipore, Darmstadt, Germany) and cultivated with the primary antibodies. After being washed with TBST, bands were incubated with relative secondary antibody for 1 h and imaged by ChemiDoc^TM^ Touch Imaging System (Bio-Rad, Hercules, CA).

**1.5 Quantitative real-time PCR (qPCR)**

Total RNAs were isolated from mice livers and different cells by Trizol reagent following manufactures instruction and quantified using NanoDrop One Microvolume UV-Vis Spectrophotometer from Thermo (Waltham, USA). Complementary DNAs (cDNA) were synthesized using HiScript III RT SuperMix kit according to the manufacturer’s specifications. The mRNA levels of targeted genes were determined by qPCR as previously described. List of used primers was provided in **Supplementary Table S1**.

**
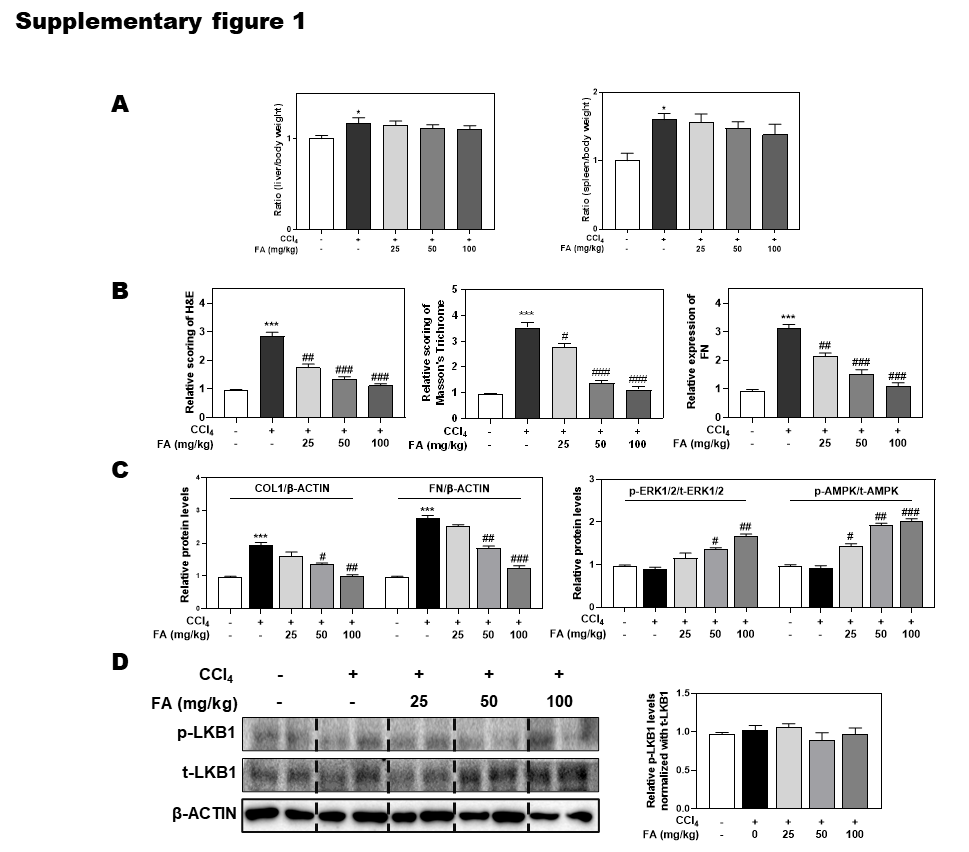
2. Supplementary figure legends**

**Supplementary Figure 1. Effects of FA on CCl_4_-induced liver injury in mice.** (**A**) Ratio of liver and spleen to body weight. (**B**) Relative scoring of H&E, Masson’s Trichrome and FN staining. (**C**) The relative density of COL1/β-ACTIN, FN/β-ACTIN, p-ERK1/2/t-ERK1/2 and p-AMPK/t-AMPK. (**D**) Representative immunoblots against p-LKB1, t-LKB1 and β-ACTIN were shown. Statistical significance: *P<0.05, ***P<0.001, compared with control group; ^#^P<0.05, ^##^P<0.01, ^###^P<0.001, compared with CCl_4_ group. One-way ANOVA with Tukey’s post-hoc tests (n=6).

**
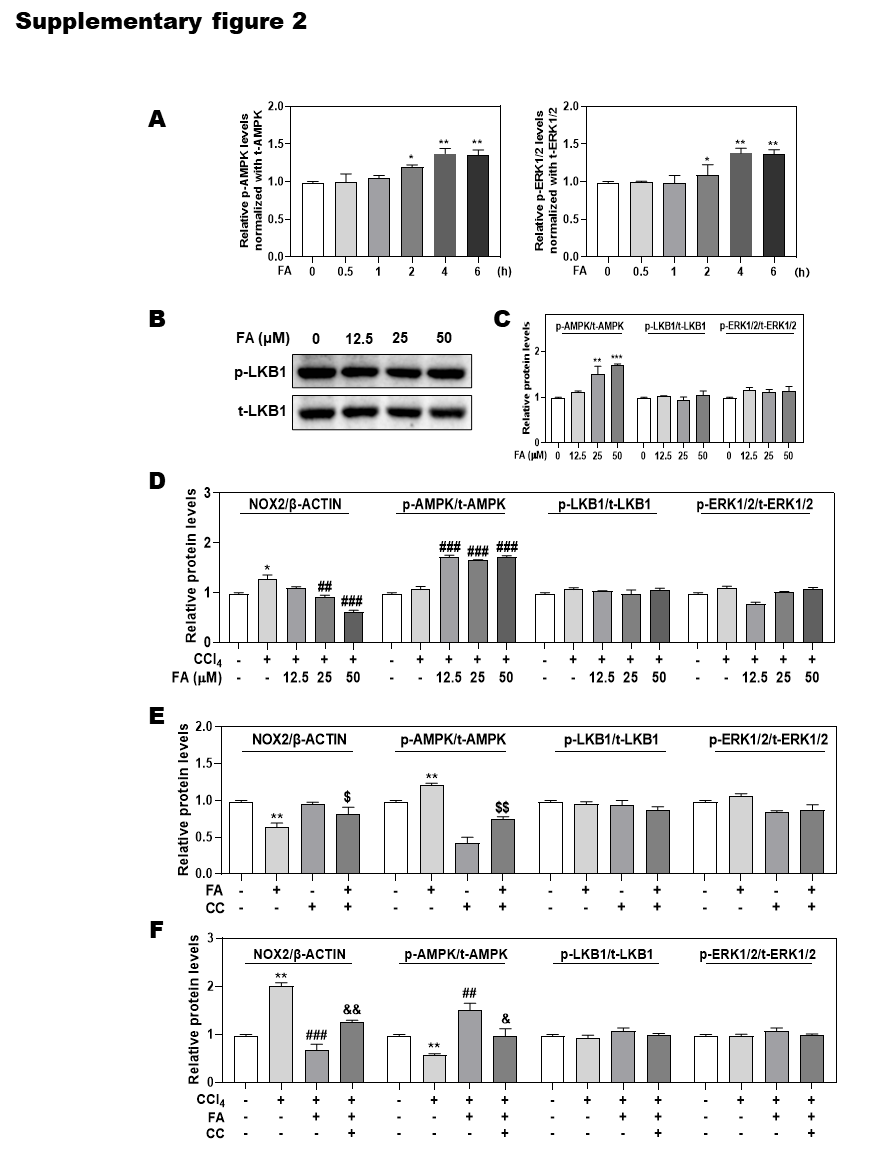
**

**Supplementary Fig. 2. Statistical analysis for western blot in MPHs.** (**A**, **C**-**F**) The relative density of p-AMPK/t-AMPK, p-ERK1/2/t-ERK1/2, p-LKB1/t-LKB1 and NOX2/β-ACTIN. (**B**) Representative immunoblots against p-LKB1 and t-LKB1 were shown. Statistical significance: *P<0.05, **P<0.01, ***P<0.001, compared with control group; ^##^P<0.01, ^###^P<0.001, compared with CCl_4_ group; ^$^P<0.05, ^$$^P<0.01, compared with FA group; ^&^P<0.05, ^&&^P<0.01, compared with CCl_4_ + FA groups. One-way ANOVA with Tukey’s post-hoc tests (n=3).

**
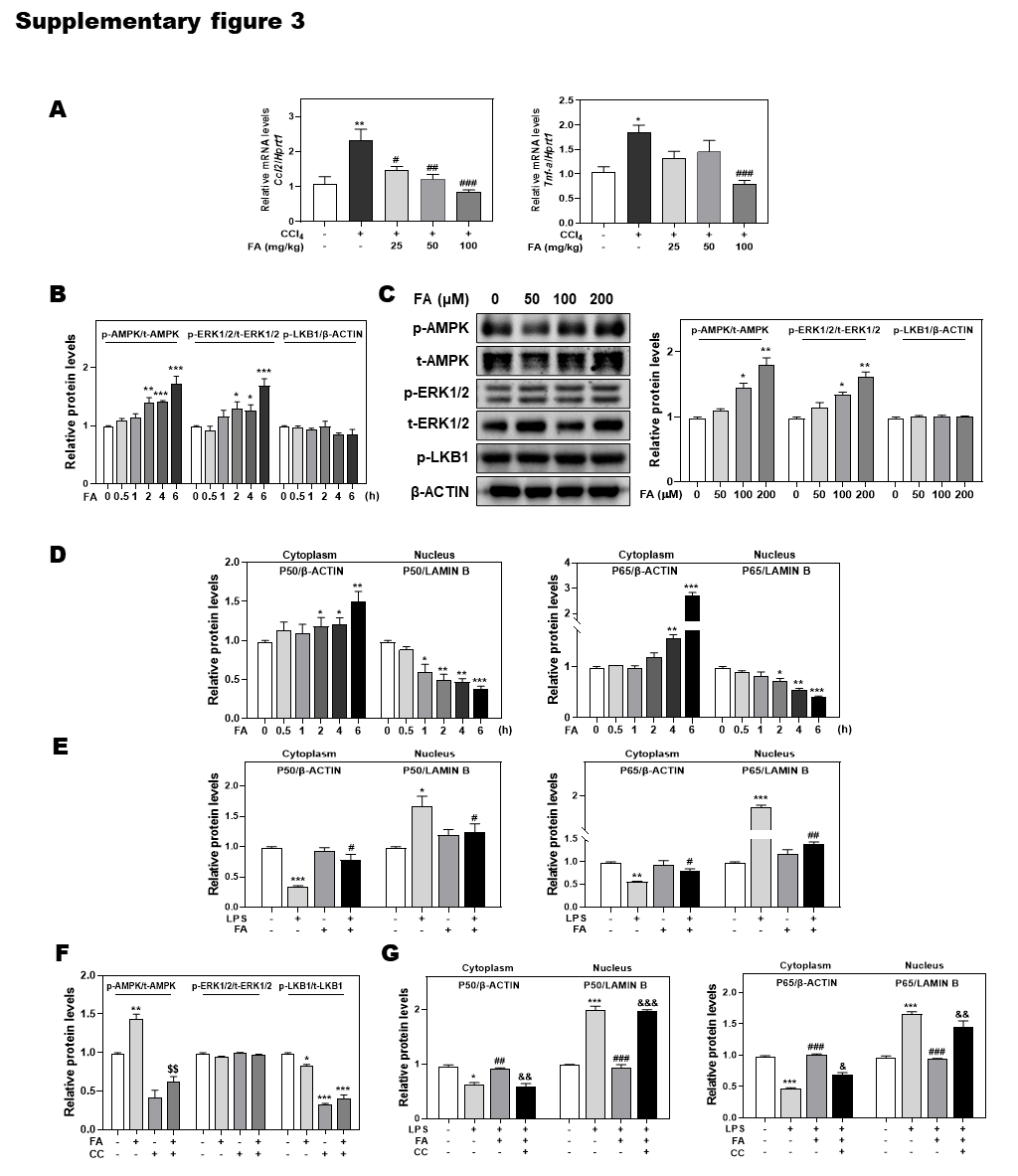
**

**Supplementary Fig. 3. Statistical analysis for western blot in RAW cells.** (**A**) Relative mRNA levels of *Ccl2* and *Tnfa* were determined by qPCR and normalized using *Hprt1* as an internal control. (**B**, **C** and **F**) The relative density of p-AMPK/t-AMPK, p-ERK1/2/t-ERK1/2 and p-LKB1/β-ACTIN. (**C**) Representative immunoblots against p-AMPK, t-AMPK, p-ERK1/2, t-ERK1/2, p-LKB1 and β-ACTIN. (**D**, **E** and **G**) The relative density of P50/β-ACTIN, P50/LAMIN B, P65/β-ACTIN and P65/LAMIN B. Statistical significance: *P<0.05, **P<0.01, ***P<0.001, compared with control group; ^#^P<0.05, ^##^P<0.01, ^###^P<0.001, compared with LPS group; ^$$^P<0.01, compared with FA group; ^&^P<0.05, ^&&^P<0.01, ^&&&^P<0.001, compared with LPS + FA groups. One-way ANOVA with Tukey’s post-hoc tests (n=3).

**
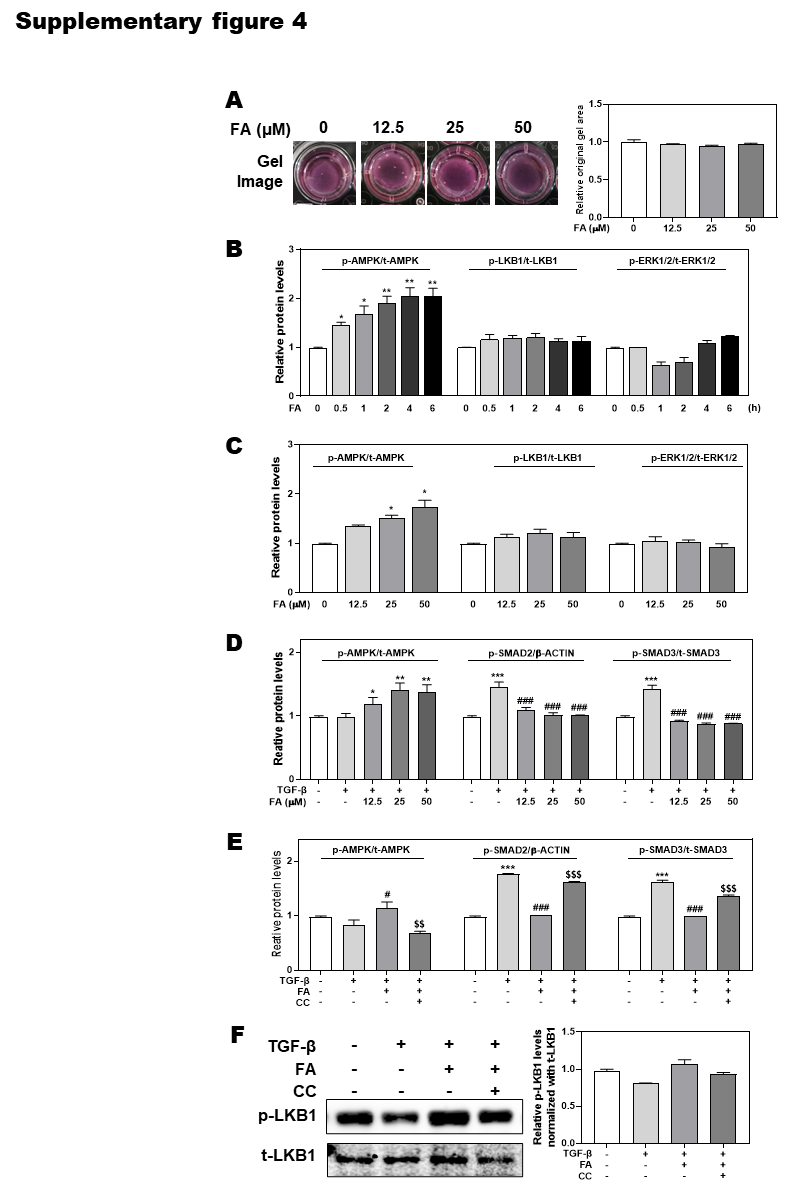
Supplementary Fig. 4. Effects of FA on gel contraction and statistical analysis for western blot in LX-2 cells*.*** (**A**) The area of collagen gel in LX-2 cells was imaged and analyzed. (**B**-**F**) The relative density of p-AMPK/t-AMPK, p-LKB1/t-LKB1, p-ERK1/2/t-ERK1/2, p-SMAD2/β-ACTIN and p-SMAD3/t-SMAD3. (**F**) Representative immunoblots against p-LKB1 and t-LKB1 were shown. Statistical significance: *P<0.05, **P<0.01, ***P<0.001, compared with control group; ^#^P<0.05, ^###^P<0.001, compared with TGF-β group; ^$$^P<0.01, ^$$$^P<0.001, compared with TGF-β + FA groups. One-way ANOVA with Tukey’s post-hoc tests (n=3).


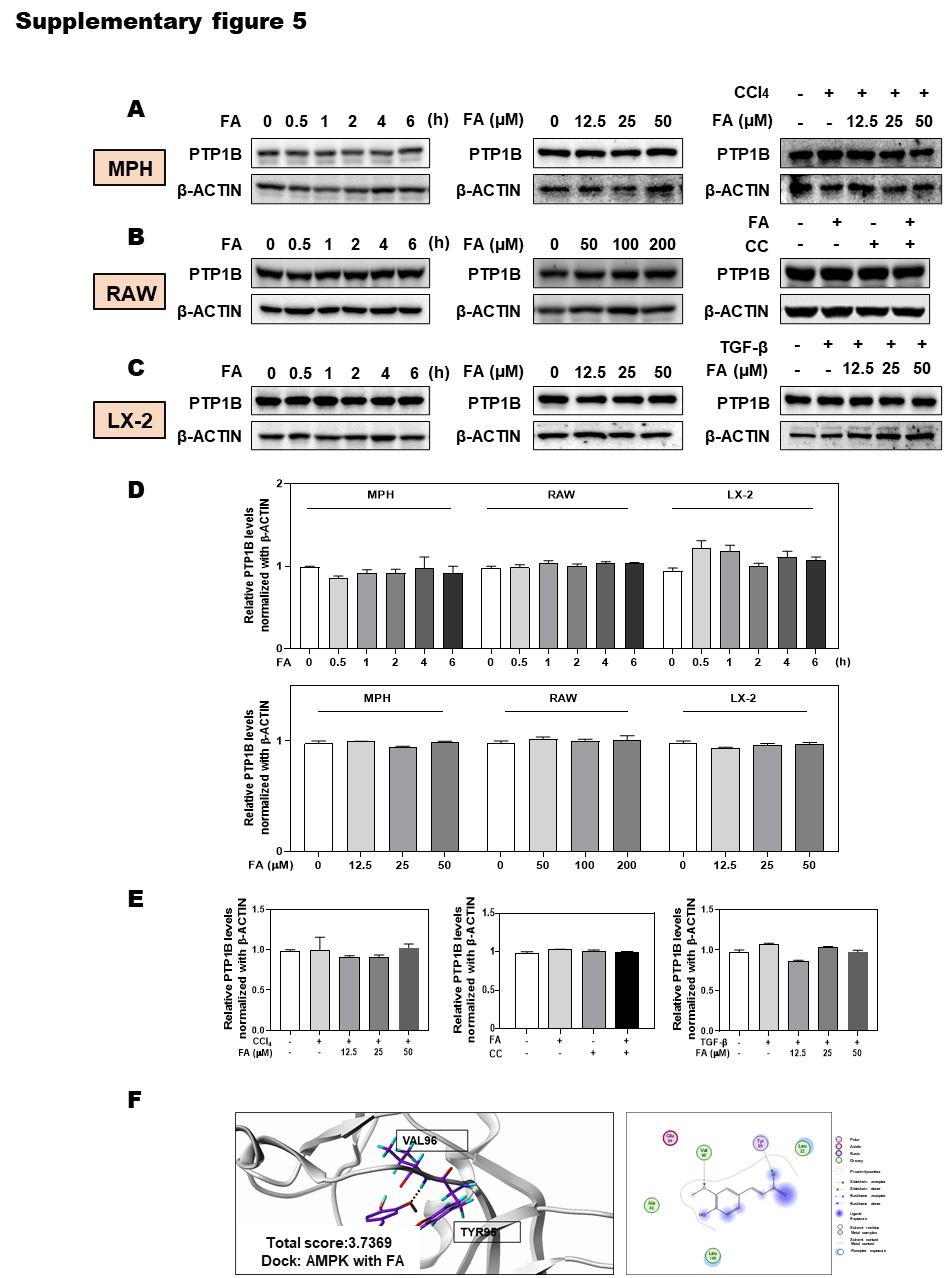
**Supplementary Fig. 5. Effects of FA on PTP1B protein expression in different liver cells and molecular docking results for FA-AMPK.** (**A**-**C**) Representative immunoblots against PTP1B and β-ACTIN were shown. (**D** and **E**) The relative density of PTP1B/β-ACTIN. (**F**) Representative images for the binding mode of FA with the crystal structure of AMPK. All experiments were conducted three times.


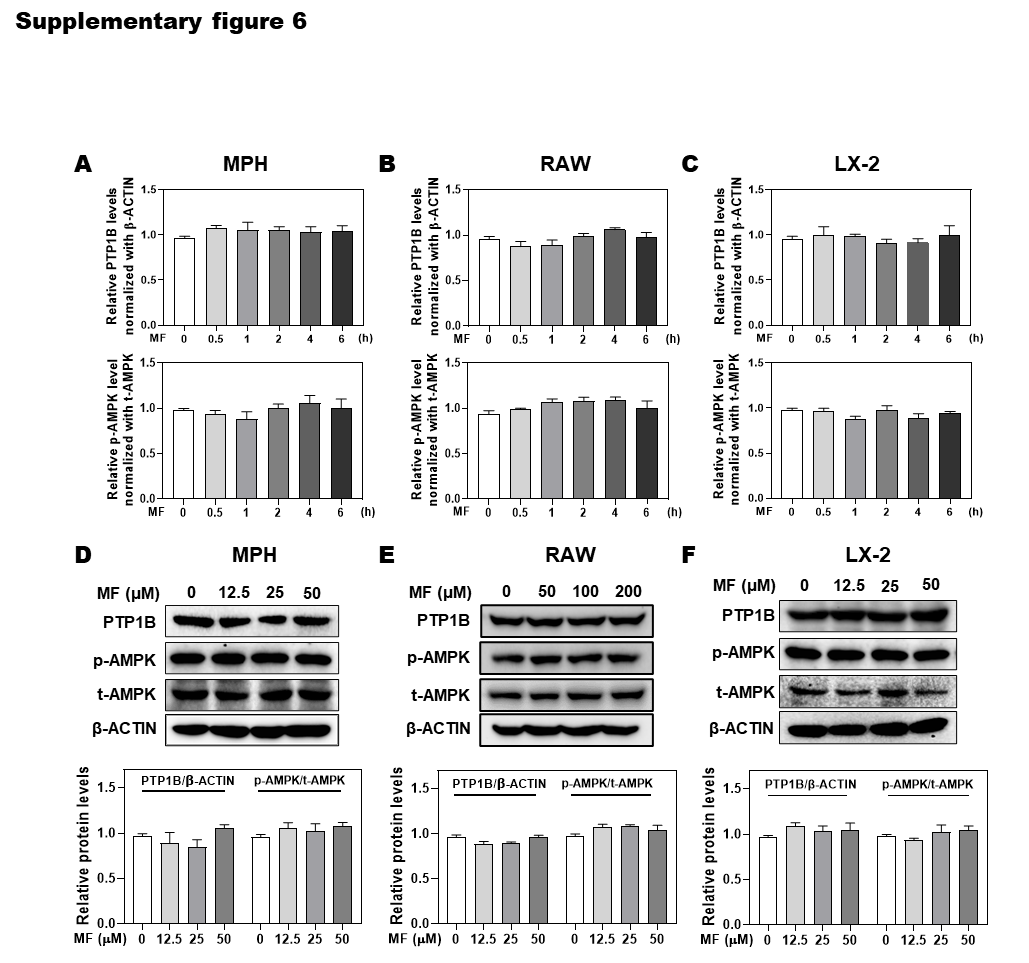
**Supplementary Fig. 6. Effects of MF on PTP1B and AMPK** **signaling pathways in different liver cells.** (**A**-**F**) The relative density of PTP1B/β-ACTIN and p-AMPK/t-AMPK. (**D**-**F**) Representative immunoblots against PTP1B, p-AMPK, t-AMPK and β-ACTIN were shown. All experiments were conducted three times.

| **Supplementary Table 1. Primer sequences used in qPCR.**  **A. Mice** | | | | |
| --- | --- | --- | --- | --- |
| **Genes** | **Forward primer (5’-3’)** | | **Reverse primer (5’-3’)** | |
| *Acta2* | GTCATCCACAGACAGAGTAGG | | CTCCCAACAGACCTGTCTATAC | |
| *Ccl2* | TCCACAACCACCTCAAGCACTTC | | GGCATCACAGTCCGAGTCACAC | |
| *Cd11b* | CGGTAGCATCAACAACAT | | GCATCAAAGAGAACAAGGT | |
| *Col1a1* | TGAACGTGGTGTACAAGGTC | | CCATCTTTACCAGGAGAACCAT | |
| *F4/80* | TGGTATGTCTTGCCTTGG | | TGGTTGTCAGTCTTGTCTAT | |
| *Fn* | CTATAGGATTGGAGACACGTGG | | CTGAAGCACTTTGTAGAGCATG | |
| *Hprt1* | CAGACTTTGTTGGATTTGAAA | | GCTCATCTTAGGCTTTGTAT | |
| *Il1b* | AATCTCACAGCAGCACATC | | AGCAGGTTATCATCATCATCC | |
| *Il6* | CTCCCAACAGACCTGTCTATAC | | CCATTGCACAACTCTTTTCTCA | |
| *Inos* | ACTCAGCCAAGCCCTCACCTAC | | TCCAATCTCTGCCTATCCGTCTCG | |
| *Tgfb1* | GACCTCAAGAGCTCTAACATCC | | GTCATCCACAGACAGAGTAGG | |
| *Tnf-a* | GAGAGAAAGTGAGTGCGTCCCTTG | | GGCAACAGCACCGCAGTACC | |
| **B. Human** |  | |  | |
| **Genes** | **Forward primer (5’-3’)** | **Reverse primer (5’-3’)** | |  |
| *Acta2* | TGAGAAGAGTTACGAGTT | CATTGTTAGCATAGAGGTC | |  |
| *Col1a1* | GGCTCCTGCTCCTCTTAG | GGATGTCTTCGTCTTGGC | |  |
| *Fn* | CTATAGGATTGGAGACACGTGG | CTGAAGCACTTTGTAGAGCATG | |  |
| *H19* | CAGGAGTGATGACGGGTGGAG | CTTCTTTCCAGCCCTAGCTCA | |  |
| *Hprt1* | TATGGCGACCCGCAGCCCT | CATCTCGAGCAAGACGTTCAG | |  |
| *Tgfb1* | CTCTCCGACCTGCCACAGA | AACCTAGATGGGCGCGATCT | |  |
